# Facilitating Domestic and Civic-Style Activity in the Later Life of Army Veterans: The Influencing Culture of the Royal Hospital Chelsea

**DOI:** 10.3390/geriatrics9050121

**Published:** 2024-09-15

**Authors:** Helen Cullen, Alison K. Osborne, Matthew D. Kiernan, Gemma Wilson-Menzfeld

**Affiliations:** 1 Department of Nursing, Midwifery, and Health, Faculty of Health and Life Sciences, Northumbria University, Newcastle upon Tyne NE7 7XA, UK; drhelencullen@gmail.com (H.C.); matt.kiernan@northumbria.ac.uk (M.D.K.); 2 Department of Psychology, Northumbria University, Newcastle upon Tyne NE1 8ST, UK; alison.osborne2@northumbria.ac.uk

**Keywords:** Chelsea Pensioner, civic engagement, interventions, veteran, ageing in place, healthy ageing, quality of life

## Abstract

The Royal Hospital Chelsea has been home to veterans of the British Army since 1692. Opportunities to remain physically active throughout the life course of its residents include participation in numerous hobbies within the quasi-military environment, and in the civic engagement representational role of the Chelsea Pensioner. This study examines the influence the Royal Hospital Chelsea culture has on resident opportunities to remain active. A non-traditional mixed-methods convergent design was used across three participant groups. Staff and established residents engaged in semi-structured qualitative interviews, with established residents and a cohort of new residents completing Quality of Life questionnaires. The findings indicate established Chelsea Pensioners experienced a sense of pride and purpose, elevated social status, and increased life satisfaction as a result of engaging in multiple activities. New Chelsea Pensioners demonstrated a trend towards increased quality of life after six months’ residence at the Royal Hospital Chelsea. Further research is required to explore the transferability of similar interventions into other residential establishments.

## 1. Introduction

Evidence indicates that recreational activity and social engagement influence experiences in later life, resulting in benefits that include feeling useful, valued, and needed [1,2]. Expanding recreational pastimes to include regular physical activity is found to contribute towards an individual’s ability to age successfully, or ‘age well’ [3,4,5,6].

The value of being socially active is of equal significance whether an individual is in mid or later life; however, opportunities can be impacted by life events such as bereavement, physical decline, or retirement from work, causing alternative activities to be identified to maintain socially active engagement [2]. Furthermore, environments which offer security, access to activities and the natural environment are found to have a positive influence on ageing ‘well’ and health status [7]. Rowe and Khan’s [6] (p. 433) theory of ageing successfully indicates a requirement for individuals to maintain *“low probability of disease and disease-related disability; high cognitive and physical function capacity; and active engagement with life”.* However, challenges to interpret the subjective term ‘successful’ led Ballesteros [3] to determine multiple interpretations collectively as ‘ageing well’.

As people age, personal circumstances and health status can change, resulting in a need for some to consider living in a place that best suits their requirements to enable them to remain living safely, independently, and physically active as possible [8]. Research within the United Kingdom (UK) suggests that the majority of older people prefer to live within their own home for as long as possible, either independently or with an assisted care package [9]. For those who choose to live independently, influences on positive well-being include living in a safe and secure location, in accommodation that may have adaptations to support individuals as they age, and with close proximity to an existing social network [10].

The concept of ‘ageing in place’ enables people to age within a place they call home, while having access to appropriate support services, and is widely recognised and implemented [11,12,13]. However, the phrase is considered to be outdated as people should be able to age in a place of their choosing, or the ‘right’ place rather than the historical ‘home’ to recognise the flexibility of the physical location individuals live in as they age [12,14].

For those older people who make the decision to move from their traditional home environment into more supported housing, the UK offers multiple residential options including sheltered housing [15], residential care homes [16], retirement villages [17], extra care housing [18], nursing homes [19], and almshouses [20]. In addition, those who have served in the UK armed forces have access to veteran-specific residential options that recognise their military service and are, as a result, tailored to meet their needs [21,22,23,24,25]. However, there is minimal research that evidences the impact that living in these establishments has on veteran health and social care outcomes.

The largest veteran-specific residential establishment in the UK is the Royal Hospital Chelsea. Established in 1692, in buildings specifically designed to reflect quasi-military surroundings, it provides a home to approximately 300 former soldiers of the British Army, known as Chelsea Pensioners, or ‘In-Pensioners’. The Royal Hospital Chelsea living environment comprises a blend of accommodation options outlined earlier, with each being available to In-Pensioner residents depending on need. Embedded within the fabric of the Royal Hospital Chelsea is a general practitioner-led medical practice [26], and an on-site independently regulated nursing home, the Margaret Thatcher Infirmary, offering nursing care for those In-Pensioners with additional care needs [27,28]. These facilities provide a home for the In-Pensioners from the time of admission to the end of their lives, enabling them to age in the ‘right’ place.

In contrast to non-veteran-specific residential establishments, those who live at the Royal Hospital Chelsea must have served in the British Army, be 66 years of age or older, or be receiving a UK Government State Pension, be free of any financial obligations to dependents, and at the point of moving into the Royal Hospital Chelsea, be able to live independently [29]. A male-only residence since 1692, the Royal Hospital Chelsea has accepted female Army veterans since 2009 [30].

In-Pensioners live in single-person ‘berths’ comprising a bedroom, small living space and bathroom, in accommodation wings known as long wards [30]. The long wards are divided into four military-style ‘companies’ with each supported by a Captain who is responsible for the welfare and conduct of In-Pensioners [31]. The role of Captain is fulfilled by former members of the British Army, alongside others who occupy quasi-military roles within the Royal Hospital Chelsea, including a Regimental Sergeant Major and Quartermaster [31]. Exceptionally, In-Pensioners are required to wear military-style uniform on a day-to-day basis both within the Royal Hospital Chelsea and externally, where they are recognisable in their Scarlet uniforms [30], which sets them apart from those in similar residential establishments.

In further contrast to a non-veteran specific establishment, the Royal Hospital Chelsea is governed by a Board of Commissioners who are nominated by the UK Government’s Secretary of State for Defence and appointed by the Monarch. A Governor role, also appointed by the Monarch, is fulfilled by a former senior officer of the British Army holding the rank of 3 or 4 star General, with responsibilities that include chairing the Board of Commissioners and overseeing the running of the Royal Hospital Chelsea [32].

In addition to providing care and support to its residents, key Royal Hospital Chelsea objectives are to offer In-Pensioners opportunities to engage in activities including, but not limited to, the provision of hobby clubs, on-site allotments, a fitness suite, bowls and boules [33], and representing both the Royal Hospital Chelsea and the wider armed forces community at formal and informal events to reduce any loneliness or isolation that they may have experienced prior to moving into the Royal Hospital Chelsea [34].

The aim of this study was to examine the influence the Royal Hospital Chelsea’s culture has on the domestic and civic engagement of Chelsea Pensioners and the resultant impact this has on their health and social outcomes and quality of life.

## 2. Materials and Methods

This paper is part of a wider study [33] that aimed to gain an understanding of the Royal Hospital Chelsea model of care and its influence on In-Pensioner health and social care outcomes. Data collection for the wider study used a non-traditional mixed-methods convergent design carried out over one phase comprising four data collection elements across three participant groups, namely key staff, In-Pensioner and new In-Pensioner cohorts.

Primary data collection comprised of quantitative measures in the form of semi-structured interviews for the key staff and In-Pensioner cohorts, with new In-Pensioners and In-Pensioners completing quantitative measures in the form of two Quality of Life questionnaires. Qualitative data collection assumed dominance as the study was explorative due to the lack of available evidence relating to the Royal Hospital Chelsea model of care. Quantitative data sought to evidence any impact living at the Royal Hospital Chelsea had on In-Pensioner and new In-Pensioner quality of life and provide a different perspective from that of the qualitative data.

Triangulating the findings from all data led to an understanding of the elements required to enable individuals to age well and in [the right] place, meeting the study aims. Qualitative and quantitative data were analysed separately ahead of triangulation.

### 2.1. Participants

Twenty-five key staff were invited to take part in the project with a total of 19 key staff recruited. Participation was voluntary and all within this cohort were Royal Hospital Chelsea employees and were identified by their job role to ensure a balanced cross-section of those employed in governance, strategic, management, and operational roles. Key staff took part in quantitative data collection only.

In-Pensioner participants lived independently within the Royal Hospital Chelsea, had all served in the British Army, were 65 years of age or older, and were selected based on duration of residence, the regiment in which they had served, and which area, or ‘long ward’, they lived in within the Royal Hospital Chelsea. A total of 25 In-Pensioner participants engaged in qualitative and quantitative data collection.

All new In-Pensioner residents had moved into the Royal Hospital Chelsea during a 12-month period (May 2021–May 2022) and were automatically invited to take part in the study. A total of 17 new In-Pensioners participants engaged in quantitative data collection only.

### 2.2. Qualitative Data Collection

Qualitative data was collected using semi-structured interviews with key staff and In-Pensioners. Interview questions sought to elicit feedback on life at the Royal Hospital Chelsea, including opportunities to, and the impact of, accessing healthcare support, the social care provision including engaging in activities, the commitment of In-Pensioners to represent the Royal Hospital Chelsea, and the impact of living and working in a historical environment alongside barriers and challenges to service delivery.

Areas for discussion were primarily the same for both key staff and In-Pensioner cohorts, which enabled responses to be considered together to provide an overall picture of the Royal Hospital Chelsea experience. The interview schedules can be found at Appendix A. Interviews were audio recorded with a Dictaphone, before being transcribed. Interview durations ranged from 60 to 120 min.

### 2.3. Quantitative Data Collection

Quantitative data collection for this paper comprised the WHOQOL-BREF (World Health Organisation Quality of Life BREF) [35] questionnaire. The WHOQOL-BREF captures quality of life data within four domains, namely physical health (Domain 1); psychological (Domain 2); social relationships (Domain 3); and environment (Domain 4). This was used to establish an evidence baseline by capturing In-Pensioner and new In-Pensioner feedback on their quality of life experience.

In-Pensioners completed the questionnaire at the end of their interview, while new In-Pensioners completed the questionnaire at the time of moving in and again six months later. The two new In-Pensioner datasets looked to identify any noticeable differences in responses from their initial arrival at the Royal Hospital Chelsea and again six months later to indicate any improvement or decline in their quality of life, to establish a baseline of quality of life evidence for this participant group.

The established In-Pensioner dataset offered a comparator to the second set of data from the new In-Pensioner cohort. The complete datasets offered a different perspective of quality of life for both participant groups.

The findings presented in this paper will focus on areas relating to the environment and physical health domains of the WHOQOL-BREF, specifically opportunities to access activities, energy levels, and mobility, respectively, as they are relevant to the study aims (questions 14, 10, and 15).

### 2.4. Data Analysis

Both qualitative and quantitative datasets were analysed separately prior to triangulation as the data were not intended to challenge each other, but to facilitate a greater understanding of the study phenomenon.

Interview transcripts were uploaded to NVivo in order to carry out data analysis. This followed the Braun and Clarke six phases of reflexive thematic analysis methodology [36]. Quantitative data was entered into SPSS^®^ (v.29.0) statistical analysis software platform where descriptive statistics were calculated.

## 3. Results

### 3.1. Qualitative

A total of 43 participants engaged in semi-structured interviews, namely 25 In-Pensioners and 19 key staff. Two primary themes were identified, each with two sub-themes (Table 1). The culture within the Royal Hospital Chelsea comprises the sub-themes ‘military culture’, and ‘ex-military staff influence in the Royal Hospital Chelsea. The facilitation of domestic and civic activities theme comprised the sub-themes ‘access to opportunities that facilitate staying active’, and ‘the representational role of the Chelsea Pensioner’.

#### 3.1.1. Culture within the Royal Hospital Chelsea

Several factors contribute towards the quasi-military culture of the Royal Hospital Chelsea, including its historical built environment created specifically to provide a home to former soldiers, a complement of staff who have previously served in the military, and the military-style regime that is embraced by In-Pensioners.

##### Military Culture

The importance of a military culture, or ‘ethos’, was articulated by many In-Pensioners as they regarded the Royal Hospital Chelsea’s military-style environment as being one with which they were familiar. Military life can create an exclusive sense of belonging, identity, and camaraderie, with these shared experiences resulting in bonds that remain throughout an individual’s lifetime: *“[…] I mean they all say to us, ‘I wanted to come to somewhere I felt as if I was getting back into the Army and being with colleagues and friends’, so if it became so civilianised and was just care, I think you would lose that tangible thread towards the military.”* (Participant 15, staff member)

For some, the military influence was a key factor in the decision to move into the Royal Hospital, with many believing these characteristics cannot be found elsewhere: *“No argument, there isn’t another care home that comes anywhere near it. Part of that is its organisation, it’s military ethos […]”* (Participant 52, In-Pensioner, resident 9 years)

The Royal Hospital Chelsea engendered feelings of ‘coming home’ as In-Pensioners settled back into a familiar military-style environment. This brought a sense of security, and a social network In-Pensioners are used to, supporting the concept that ageing in a familiar, or ‘right’, place contributes towards positive quality of life outcomes and the ability to age ‘well’: *“It’s home. I’d go nowhere else. If I had millions of pounds, I couldn’t be any happier than I am here because I am home but then again, I grew up in the [military] system.”* (Participant 20, In-Pensioner, resident 6½ years)

Despite displaying collective traits including the prevalence of a military mindset, sense of humour, and shared military experiences, In-Pensioners considered themselves as individuals, retaining a personal identity and levels of independence: *“I think people have all got their own characteristics.”* (Participant 02, In-Pensioner, resident 3 years)

In-Pensioners considered the Royal Hospital Chelsea to be an exclusive residence which they believed instilled a sense of pride and created meaning to their lives with some indicating the decision to live within the establishment was life-changing: *“Oh, well, […] coming here and living here is a new life for me. Well, it is an old life, but it has brought my life back […]”* (Participant 73, In-Pensioner, resident 3½ years)

##### Ex-Military Staff Influence in the Royal Hospital Chelsea

In-Pensioners indicated a preference to engage with staff who had served in the military, with some believing those staff without military experience or knowledge were unable to understand the military mindset, the military-specific language they use, or the experiences they have encountered as a result of their service. The preference to engage with former military staff was recognised by many participants as being important to In-Pensioners: *“…I think it is absolutely critical that the military role is maintained. They can confide in me, they can tell me things that they won’t tell the family, so I think it is absolutely essential that that [military] background is acknowledged.”* (Participant 15, staff member)

The role of Captain was occupied by former members of the Army which resulted in an understanding of In-Pensioner personalities and characteristics. Captains provided practical daily support ranging from encouraging participation in group activities or hobby-oriented pastimes, to more specific personal well-being or peer support. This may, for example, include speaking to an In-Pensioner who may have been missing from breakfast or is thought to be behaving out of character and may therefore require assistance: *“…they may come and say to you can I speak to you in confidence, and then they will tell you something, […] it could be about another In-Pensioner, and then you would go and investigate to make sure that In-Pensioner was ok.”* (Participant 21, staff member)

Despite the clear indication that In-Pensioners enjoyed living in a military-style environment, and some preferring ex-military staff, there was an acknowledgement that a blended workforce that included ‘civilian’ staff was necessary to ensure they were supported appropriately. In addition, In-Pensioners recognised that they needed to accept the Royal Hospital Chelsea was a residence for older people, suggesting levels of conformity similar to that experienced while in military service: *“Most of us that think about it understand that as a care facility for older people, that we have to obey the rules and we need to employ people who understand the process.”* (Participant 44, In-Pensioner, resident 2 years)

#### 3.1.2. Facilitation of Domestic and Civic Activities

A fundamental element of the Royal Hospital Chelsea is the commitment to ensure In-Pensioners have access to a wide variety of activities either domestically, within the Royal Hospital Chelsea, or externally in the representational role of Chelsea Pensioner. Participation in these activities resulted in In-Pensioners expressing a sense of purpose, pride, identity, and elevated status, contributing towards positive quality of life.

##### Access to Opportunities That Facilitate Staying Active

In-Pensioners had access to a multitude of activities within the Royal Hospital Chelsea, ranging from sedentary to more energetic options dependent on personal choice and physical ability, which improved social connectivity with fellow residents, and fostered a sense of purpose: *“I wrote a few down here, whist, crib, pottery, choir, pace sticking, which is a military thing, fishing, library […] that is just a few of the things I can think of off the top of my head. I mean, you can take part in as many or as little as you want.”* (Participant 46, In-Pensioner, resident 3 years)

In-Pensioners demonstrated enthusiasm and determination to remain active within the Royal Hospital Chelsea, and maintain social engagement with fellow In-Pensioners, staff, and the wider community, even if reduced mobility as a result of ageing or decline in physical ability restricted their ability to do so: *“Well, there is lots of activities going on if you are able. […] I put my name down for a lot of things but now, of course I am like this [less mobile], but I do what I can.”* (Participant 58, In-Pensioner, resident 6 years)

Opportunities to remain active extended to engaging in internal ‘jobs’ such as mentoring new In-Pensioners, undertaking ‘tour guide’ roles escorting visitors around the Royal Hospital Chelsea, supporting the internal postal system and accompanying fellow In-Pensioners to hospital appointments, which In-Pensioners embraced: *“Quite a lot of people have internal jobs as I do. I do mentoring and so on and so forth.”* (Participant 20, In-Pensioner, resident 6½ years)

Staff were committed to supporting In-Pensioners to engage in as many events as they chose to, which included encouraging participation in new interests and ideas, facilitating in engagement in meaningful activities, resulting in improved physical health: *“Never before have I worked in a place where you can […] say, ‘Hey, I’ve got an idea. I really want to wing walk and raise money for the Royal Hospital’, and they just go, ‘Yeah, great idea. We will help you make it happen’.”* (Participant 32, staff member)

##### The Representational Role of the Chelsea Pensioner

An integral part of living within the Royal Hospital Chelsea is the requirement that In-Pensioners represent the place in which they live for a minimum of two years by wearing a military-style uniform, referred to as the Scarlet(s), and engage in internal and external activities. The opportunity to wear uniform, representing the Royal Hospital Chelsea and wider military community, was embraced enthusiastically by all In-Pensioners irrespective of age or length of residence, with physical ability being the only evident barrier preventing participation.

The role of the uniformed Chelsea Pensioner increases In-Pensioner visibility as they experience enhanced, often celebrity-like, social status, and are afforded access to prestigious events and venues, contributing towards a sense of purpose, belonging, identity, and pride. In-Pensioner confidence increased, enabling them to engage with people they would otherwise be reluctant to speak to, subsequently increasing their social network and life satisfaction: *“When we put on our Scarlet coat it just reinforces that this is my regiment, this is my organisation, I am part of this organisation, same as guys have been for the same 300 odd years and I am proud to put that coat on and wear it. I might not say that every time, but I am sure most of us feel that.”* (Participant 44, In-Pensioner, resident 2 years)

The commitment to represent the Royal Hospital Chelsea was demonstrated through civic engagement style roles that helped raise awareness of the establishment in which they lived and its past and present residents, which facilitated proactive social engagement with members of the public, veterans, and the Armed Forces Community: *“But they don’t want to sit about. They want to be hard working. They want to be purposeful. They want to be active, and I know lots of older people want that too, but as a group here, you know, there is probably more of that […] but yeah, I think that just all comes together to make this place, you know, the special sort of melting pot that it is really.”* (Participant 32, staff member)

The extant outreach programme presented opportunities for In-Pensioners to take part in civic engagement style activities as they engaged with veterans in prisons, those in the community experiencing homelessness, and schools, further raising awareness of the Royal Hospital Chelsea.

The active civic engagement and representative positioning gave In-Pensioners a sense of purpose, reinforced their identity and engendered a sense of pride in themselves and the place in which they live, all of which contributed towards positive life satisfaction.

In-Pensioner identity and visibility was enhanced as a result of their representational role, which was considered by one participant to diminish as people grew older: *“I mean, it’s an obvious thing to say but as you get older you get more invisible and I think a lot of older people feel very, very, invisible indeed. You are not invisible as a Chelsea Pensioner […] you’re not just an old person, you are seen as somebody who contributed to your country’s safety, and you know…. I think that’s huge.”* (Participant 55, staff member)

Living within the military-style environment of Royal Hospital Chelsea, similar to that experienced while serving in the Army, fostered camaraderie and peer support, creating a sociable community embraced by In-Pensioners, which reduced loneliness and contributed towards positive well-being.

In-Pensioners recognised that their ambassadorial role as a Chelsea Pensioner was juxtaposed with those of comparable age living in other residential establishments, as the Royal Hospital Chelsea offered the ability to engage in a multitude of activities and events that they would not have access to if they lived elsewhere: *“… it does get you entry to places that I couldn’t conceivably have entered if it had not been for the fact that I was a Chelsea Pensioner and I have met people that I could not have met if I hadn’t been a Chelsea Pensioner.”* (Participant 02, In-Pensioner, resident 2 years)

### 3.2. In-Pensioner and New In-Pensioner Quality of Life

The small sample size limits the generalisability of the findings; however, the quantitative data collection for the wider study produced a baseline indicator of quality of life for new In-Pensioners, which was supplemented with data from the In-Pensioner cohort, and met the wider study aims [33].

As discussed earlier, the findings presented in this study relate specifically to areas that are relevant to the study aims. Full quantitative data results are available in the wider study [33].

Following completion and scoring of the WHOQOL-BREF, results from three questions were extracted from Domain 1: physical health and Domain 4: environment to aid further discussion around the aims of this paper (see Table 1 for means and standard deviations of scores on these questions). Only findings relevant to this study are presented in Table 2.

When considering the physical health domain, all participant groups indicated high mean scores relating to energy levels (Q10), with In-Pensioners demonstrating a slightly higher mean score (mean = 4.12, SD = 0.781) than both new In-Pensioner Part 1 (mean = 4.00, SD = 0.612) and Part 2 (mean = 4.06, SD = 0.659) mean scores, which may indicate that living at the Royal Hospital, and utilising the support available, resulted in increased energy levels.

New In-Pensioners indicated an increase in their mobility levels (Q15), with scores rising from mean = 3.94 (SD = 0.827) on arrival at the Royal Hospital to mean = 4.00 (SD = 0.935) six months later. Conversely, In-Pensioner scores were lower than both new In-Pensioner Part 1 scores (mean = 3.68, SD = 0.900) and Part 2 scores (mean = 4.0, SD = 0.935), which may be as a result of new In-Pensioners being required to live independently and represent the Royal Hospital Chelsea for a minimum of two years, and the In-Pensioner demographics with the cohort potentially being older, living longer at the Royal Hospital Chelsea, and experiencing reduced mobility levels.

Across both participant groups, established In-Pensioners demonstrated the highest levels of satisfaction relating to opportunities to access leisure activities (Q14) (mean = 4.67, SD = 0.482), suggesting that they were used to the environment in which they lived and the facilities on offer. However, the new In-Pensioner cohort indicated increased satisfaction from their initial arrival score 4.38 (SD = 0.085) to 4.53 (SD = 0.514), suggesting they were settling into their new environment and subsequently engaging in new interests.

Following one-way ANOVA tests, no statistically significant differences were observed between participant groups for scores on any of the questions.

## 4. Discussion

This study aimed to examine the influence the Royal Hospital Chelsea’s culture has on the domestic and civic engagement of Chelsea Pensioners and the resultant impact this has on their health and social outcomes and quality of life. Following semi-structured interviews with key staff and In-Pensioners alongside the completion of quality of life measures with established In-Pensioners and new In-Pensioners, a number of themes were developed indicating the role of the Royal Hospital Chelsea in ageing well and in ‘the right’ place. In contrast with other residential establishments, a quasi-military culture pervades the Royal Hospital Chelsea, which is reflected in the day-to-day practices, encouraging the military mindset, facilitating a social network and providing a sense of security. This is further supported by the employment of ex-military staff, ensuring In-Pensioners are comfortable and confident in their environment. The facilitation of domestic and civic activities, including representing the Royal Hospital Chelsea as Chelsea Pensioners, builds on this, supporting In-Pensioners in ageing well and contributing towards a sense of purpose, belonging, identity and pride.

Ageing ‘well’ and ageing in the ‘right’ place are important components of an individual’s ability to maintain good physical health and remain active for as long as possible within the most appropriate residential setting to meet an individual’s needs [6,37,38,39]. Under the heading of ‘staying active’ in the wider study that this paper is based on, these findings are one of four key components identified as being integral to In-Pensioner health and social care outcomes and the Royal Hospital Chelsea model of care [33].

### 4.1. Culture of the Royal Hospital Chelsea

#### 4.1.1. Military Culture

In-Pensioners clearly identify with the ethos of the Royal Hospital Cheslea, considering it to be a military-style environment that reflects the shared characteristics and values, or culture, experienced while serving in the Army. The connection to their previous military lives generates feelings of familiarity and comfortability that leads some In-Pensioners to describe living at the Royal Hospital as ‘returning home’. This familiarity is further strengthened as In-Pensioners reconnect with fellow residents who share the common bond of having served in the Army. The study findings reflect those of Netherland et al. [40], who identified the importance of living in familiar surroundings as it facilitates ‘positive’ ageing, enables individuals to stay connected to the place they call home, and within a community they recognise.

Connections established at the Royal Hospital Chelsea reflected the camaraderie experienced while in military service, where In-Pensioners considered each other as ‘family’, particularly those living within their immediate long ward environment. This familial regard is recognised within the military setting, as personnel learn to rely on each other for support and protection, with bonds and military cohesion developing early in, and throughout, their careers [41,42,43]. However, this contrasts with Abbott et al. [44], who found sheltered housing and residential home occupants enjoyed elements of social engagement, yet the development of friendships was limited, with evidence of adapting to their surroundings rather than becoming friends with the people they were now living with, keeping fellow residents at arm’s-length.

In-Pensioners are encouraged by staff to build existing and new friendships, which contrasts with McKee et al. [45], who suggest developing friendships within residential care settings is influenced by personal choice and the structural composition of the environment residents live in, with relationships ranging from being ‘friends’ to ‘good friends’, with both levels being influenced by engagement in activities. Furthermore, some residents preferred to remain in their rooms and were not encouraged by staff to engage with fellow residents. Interestingly, McKee et al. [45] found those who were connected through similar interests before becoming residents formed ‘good’ friendships, which it is suggested concurs with the shared military connection experienced by In-Pensioners.

The Royal Hospital Chelsea environment offers facilities akin to those of a military establishment or small village community through providing access to support services such as doctors, shops, green space, and leisure facilities, which are considered important for a positive interaction between the person and the environment, resulting in satisfaction with the place in which an individual lives [46].

When deciding which residence to live in, the importance of familiarity was highlighted by Reed et al. [47], who found residents felt connected to an establishment that was known to them. It could be argued that the awareness In-Pensioners have of the Royal Hospital Chelsea, as a result of their military service, creates levels of familiarity and attachment many years ahead of being eligible to live there, which may influence the decision to move into the Royal Hospital Chelsea, and contrasts with alternative residential options where prior knowledge may be absent. Furthermore, this familiarity may help In-Pensioners to become established within their new environment, arguably ageing in a place of their choosing, thus mitigating the resistance by some to relocate later in life, which can have a negative impact on health outcomes [48,49,50].

#### 4.1.2. Ex-Military Staff Influence in the Royal Hospital Chelsea/In-Pensioner Outcomes

Another consideration in the importance of ageing in ‘the right’ place is the ability to connect to staff. The In-Pensioners articulated the positive influence of ex-military staff, with many believing that understanding the military mindset, language, and culture, helped to break down any communication barriers and encourages engagement. The relationship between In-Pensioners and ex-military staff brought a level of reassurance and peace of mind as they felt they could communicate with people who ‘get’, or understand, them.

It could be argued that the Royal Hospital’s ex-military staffing structure resembles that of peer support workers, or ‘veterans-supporting-veterans’, which is known to improve veteran engagement and outcomes [51,52,53]. In contrast however, Weir et al. [53] found the veteran-veteran relationship presented challenges when the connections were too familiar; for example, when each party had served together, indicating potential barriers to engagement for some veterans. While it is possible the Royal Hospital ex-military staff may have served within the same regiment(s), it is suggested that the hierarchical status of the Royal Hospital quasi-military staff positions may lessen any challenges to engagement as In-Pensioners are already familiar with the ‘officer-vs-soldier’ relationship status and the boundaries this established during military service.

### 4.2. Facilitation of Domestic and Civic Activities

#### 4.2.1. Access to Opportunities That Facilitate Staying Active

Beyond the environment and relationships, it is important to consider the ‘offer’ of the Royal Hospital Chelsea regarding domestic and civic activities and how this contributes towards ageing well. Activity theory posits the importance of engaging in pursuits that generate feelings of usefulness, being needed, and contributing to society, all of which result in an increase in life satisfaction [2,54]. Furthermore, self-worth is found to increase with the degree of engagement, with higher frequency resulting in enhanced self-worth [2].

As evidenced by Guihan et al. [55], Kheirbek et al. [56], and Montross et al. [57], access to, and engagement in, activities improve general health status and contribute towards positive quality of life outcomes for individuals living in residential establishments. However, opportunities to stay active may be constrained by the built environment, which may itself represent a barrier, as people consider residential establishments to be institutions that are isolated from the ‘outside world’, are therefore impenetrable, and not welcoming of visitors [58]. Furthermore, those who live in institutional settings may be disadvantaged as a lack of resources may restrict opportunities for residents to participate in activities [59].

The study findings indicate that In-Pensioners are afforded access to a variety of social activities, with many participants eager to articulate the availability of numerous pastimes, ranging from sedentary card and board games through to more physically engaging activities such as hobby clubs, gardening and growing vegetables, fitness room, bowling and boules clubs, alongside an on-site café and the Chelsea Pensioner Club to facilitate social engagement [33]. Being socially engaged and taking part in meaningful activities are positive contributors that enhance an individual’s life experience as they age [60].

In-Pensioners are encouraged to stay active and are supported to push the boundaries of possibilities, reflecting the Royal Hospital Chelsea’s proactive approach to encouraging In-Pensioners to engage in meaningful activities, which is found to contribute towards ageing well [3]. Furthermore, the findings clearly evidence the strong desire by In-Pensioners to remain active participants of Royal Hospital Chelsea life even if they are physically restricted. To enable this, In-Pensioner access to, and engagement in, activities is a fundamental component of life at the Royal Hospital Chelsea and is one that is facilitated by a bespoke social care team, who work alongside other members of staff, including those in quasi-military roles, to ensure optimal engagement by In-Pensioners in activities of their choosing and within their personal capabilities, minimising the risk of isolation or loss of independence and enhancing their life satisfaction.

In contrast to the Royal Hospital Chelsea, Cook et al. [61] (2015) found that despite the willingness of sheltered housing residents to engage in activities, and demonstrating the desire to remain healthy and enjoy their lives, a lack of provision limited their opportunities to do so. In addition, a lack of financial and staff resources is found to impact on the ability to provide activities for those living in residential establishments [5,59,62].

Furthermore, Smith et al. [62] who, when exploring the impact of engaging in meaningful activity in care homes in England, found that provision of activities and encouraging resident engagement was seen as separate to the core objective of providing care and support for residents, even though residents indicated a desire to engage in activities within and external to their residence.

In-Pensioner satisfaction with opportunities to engage in activities is supported by high scores in the quality of life data, and while New In-Pensioners indicated a trend towards increased quality of life after six months’ residence, there are challenges when comparing these findings with those of established In-Pensioners, as variables including age, duration of residence, and their health status may have influenced In-Pensioner responses.

Participating in meaningful activities positively influences opportunities to age well, [3] as reflected in the study findings; however, in other contexts, research has found that both residents and staff assumed that those with declining health or ability were more accepting of engaging in fewer activities [62]. This conclusion may be as a result of over-worked staff who prioritised practical elements of resident care over opportunities to engage in activities; however, studies also suggest that those living in residential care are not physically active [63]. Health guidelines suggest activity levels for those over 65 years of age should include daily light activity such as walking slowly, or undertaking light household tasks, alongside a weekly activity routine of a minimum of two and a half hours of ‘moderate intensity’ activity such as walking at pace, or dance classes, or a minimum of one hour and fifteen minutes of ‘vigorous intensity’ activity such as going for a run, swim, or fast bike ride [64,65]. Health policy proposes “activity and exercise which improve physical health, increase the sense of well-being and also tend to promote more positive social interaction and will in turn promote positive mental health” [66] (p. 110); however, evidence suggests a decline in activity levels as we age, with 10% of males and 20% of females older than 75 years of age considered to be below the threshold of good health due to inactivity [67].

In-Pensioners clearly indicate a desire to be proactive and were enthusiastic in their approach to engaging in activities, which juxtaposes with Brownie and Horstmanshof [60], who found residents experienced levels of inactivity and boredom, with long periods of time spent in their own rooms, or within communal areas watching television, which can lead to a loss of meaning in life, a loss of independence, and an increase in helplessness.

#### 4.2.2. The Representational Role of the Chelsea Pensioner

Activities may not be exclusively physical in the traditional sense. At the Royal Hospital Chelsea, In-Pensioners are encouraged to establish new roles that help to create a sense of purpose and identity, with some indicating living at the Royal Hospital Chelsea has fostered a new lease on life, creating a new purposeful element to their lives that some indicate had been lost. This contrasts with the findings of Anderson and Dabelko-Schoeny [59], and Kruse and Schmitt [5], who found those living in residential establishments can be overlooked for engagement in meaningful activities simply as a result of their age.

In-Pensioners demonstrated an enthusiasm to voluntarily engage in numerous internal roles, which included helping in the post-room, the library, and on-site shop, or as tour guides where visitors are escorted around the Royal Hospital Chelsea to learn about the history of the Chelsea Pensioners and the built environment. Findings indicate this enthusiasm and willingness to engage also extends to the In-Pensioner external-facing ambassadorial role.

In addition to the recreational and physical activities In-Pensioners take part in, they undertake the representational role of Chelsea Pensioners which, it is argued, aligns with that of civic engagement, where individuals willingly engage with others in activities that may include volunteering, community events, and educational engagement, to benefit wider society [5,59,68]. However, the benefits older people bring to society as a result of this engagement, particularly those over 80 years of age, are often overlooked by the public. Furthermore, this area is under-researched by academia, with assertions that this age group are not expected to take part in such engagements due to their increasing age and frailty [5].

Benefits to engaging in civic-style engagements can include an increase in both physical and psychological outcomes, as individuals recognise their own self-worth, enhance their ability to engage with others, develop a sense of belonging, enjoy life more, and potentially live longer [5,44,59,69]. Despite these benefits, Anderson and Dabelko-Schoeny [59] indicate there is a lack of evidence relating to opportunities for, and the impact of, engaging in civic engagement style activities for those living in residential establishments.

Volunteer-style engagement and participating in social activities is found to increase satisfaction with life, create a sense of purpose and be a positive indicator towards living longer [68,69,70]; however, this impact is found to decrease in those over 85 years of age [69]. As indicated in the study findings, it could be suggested that In-Pensioners experience these positive outcomes as a result of living at, and representing, the Royal Hospital Chelsea.

Being a Chelsea Pensioner, wearing the Scarlets, and representing the Royal Hospital Chelsea, situates In-Pensioners in an exclusive position which facilitates opportunities for them to engage in many activities and events that they would not have access to if they lived elsewhere.

### 4.3. Strengths and Limitations

This study has several strengths and limitations for consideration.

The inclusion of both Key Staff and In-Pensioner participants strengthened the study as opportunities to explore the views of both groups ensured a balanced opinion of the Royal Hospital Chelsea model of care, from both service provider and service user perspectives. Furthermore, these opinions were analysed as one dataset to facilitate a focus on the model of care rather than a ‘compare and contrast’ presentation of the findings between the participant cohorts.

The small sample sizes across all participant groups make the transferability of the results challenging, therefore consideration should be given when making comparisons with other findings.

The small quantitative data sample size limits the generalisability of the quality of life findings. Collectively, 42 In-Pensioners and new In-Pensioners completed questionnaires; however, in isolation, numbers were 25 and 17 respectively. Several elements need to be considered when determining sample sizes [71] and for this study, qualitative data assumed primacy and data collection methods were different for each participant cohort. Small In-Pensioner and new In-Pensioner participant numbers determined the amount of quantitative data available; however, the maximum amount of data possible was collected from these two participant groups. Furthermore, the quantitative findings aimed to create a quality of life evidence baseline to facilitate further data gathering in the wider In-Pensioner population; therefore, current data findings are arguably more relevant to the Royal Hospital Chelsea than other residential establishments.

Caution should be applied when comparing new In-Pensioner and In-Pensioner quantitative results as In-Pensioner variables including age, duration of residence, and potential declining health and mobility status may have influenced their feedback. This contrasts with new In-Pensioners, who were required to be able to live independently and represent the Royal Hospital Chelsea for a minimum of two years and may therefore arguably be younger and fitter at the time of admission.

## 5. Conclusions

The Royal Hospital Chelsea offers In-Pensioners opportunities to engage in domestic activities in the form of hobbies or interests within their home environment and presents a unique opportunity to represent the establishment they call home, in the ambassadorial civic-style role of Chelsea Pensioner. Collectively, these activities enable In-Pensioners to be socially interactive, and develop relationships with others that cross the generational divide, while maintaining their independence, visibility, and relevance, which are all important factors in the ability to age well, and arguably in the ‘right’ place.

In-Pensioner outcomes are influenced by the support of ex-military staff employed in quasi-military roles; however, a blended workforce ensures a holistic approach to the support offered to In-Pensioners.

Arguably, opportunities for In-Pensioners to participate in a variety of activities available as a direct result of living at the Royal Hospital Chelsea may be considered an intervention that encourages and supports lifelong engagement in activities that are commensurate with an individual’s physical ability.

The study findings contribute towards closing the gap in evidence relating to the impact of remaining active within residential establishments, and moreover by engaging in civic-style engagements. However, more research is needed to further evidence the impact similar activities may have on the health and social outcomes of those living in veteran-specific and other residential establishments. Future research that includes a control group and veteran-specific, or other specific employment type of residential establishment, may further inform the evidence base.

## Figures and Tables

**Table 1:** Qualitative themes.

Primary Theme	Sub-Themes
The culture within the Royal Hospital Chelsea	Military culture Ex-military staff influence in the Royal Hospital Chelsea
The facilitation of domestic and civic activities	Access to opportunities that facilitate staying active The representational role of the Chelsea Pensioner

**Table 2:** WHOQOL-BREF Domain 1: physical health and Domain 4: environment extracts.

WHOQOL-BREF Domain 1: Physical Health and Domain 4: Environment (extracts)				
		Domain 1: Physical Health		Domain 4: Environment
Participant Group		Q10 Do you have enough energy for everyday life?	Q15 How well are you able to get around physically?	Q14 To what extent do you have the opportunity for leisure activities?
**In Pensioner** **(Established)**	Mean	**4.12**	**3.68**	**4.67**
	N	25	25	24
	Std. Deviation	0.781	0.900	0.482
**New In-Pensioner** **Part 1**	Mean	**4.00**	**3.94**	**4.38**
	N	17	17	16
	Std. Deviation	0.612	0.827	0.885
**New In-Pensioner** **Part 2**	Mean	**4.06**	**4.00**	**4.53**
	N	17	17	17
	Std. Deviation	0.659	0.935	0.514

WHOQOL-BREF Domains: Domain 1: physical health; Domain 2: psychological; Domain 3: social relationships; Domain 4: environment.

**Figure A1:**
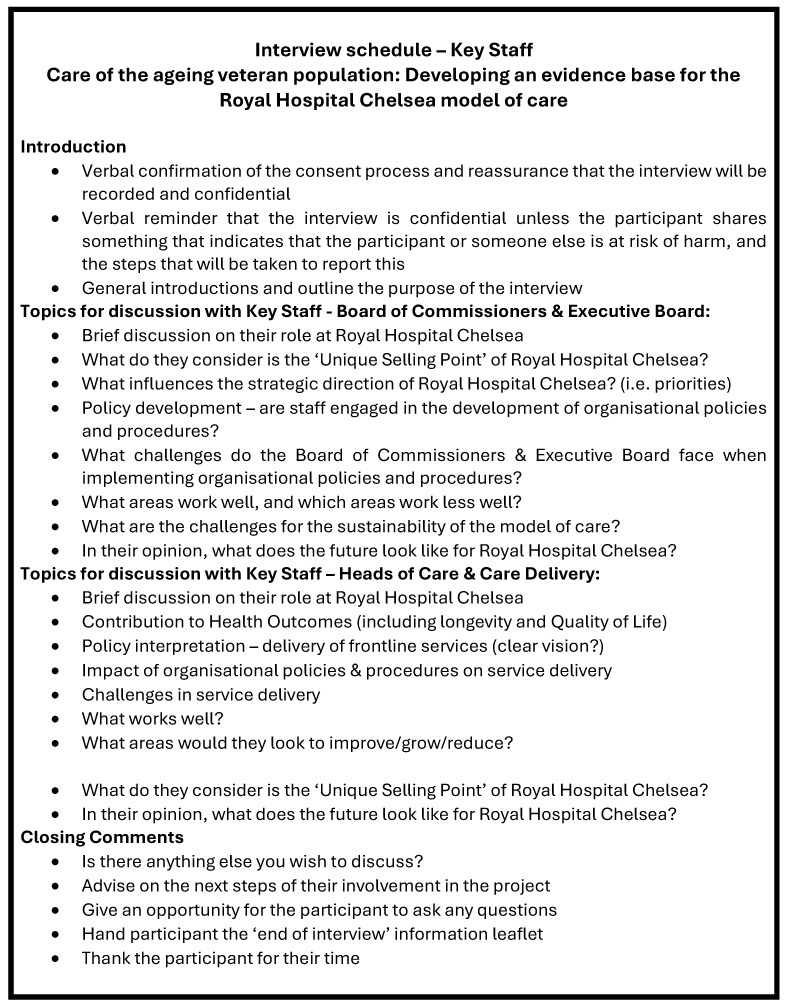
Interview schedule—Key staff care of the ageing veteran population: Developing an evidence base for the Royal Hospital Chelsea model of care.

**Figure A2:**
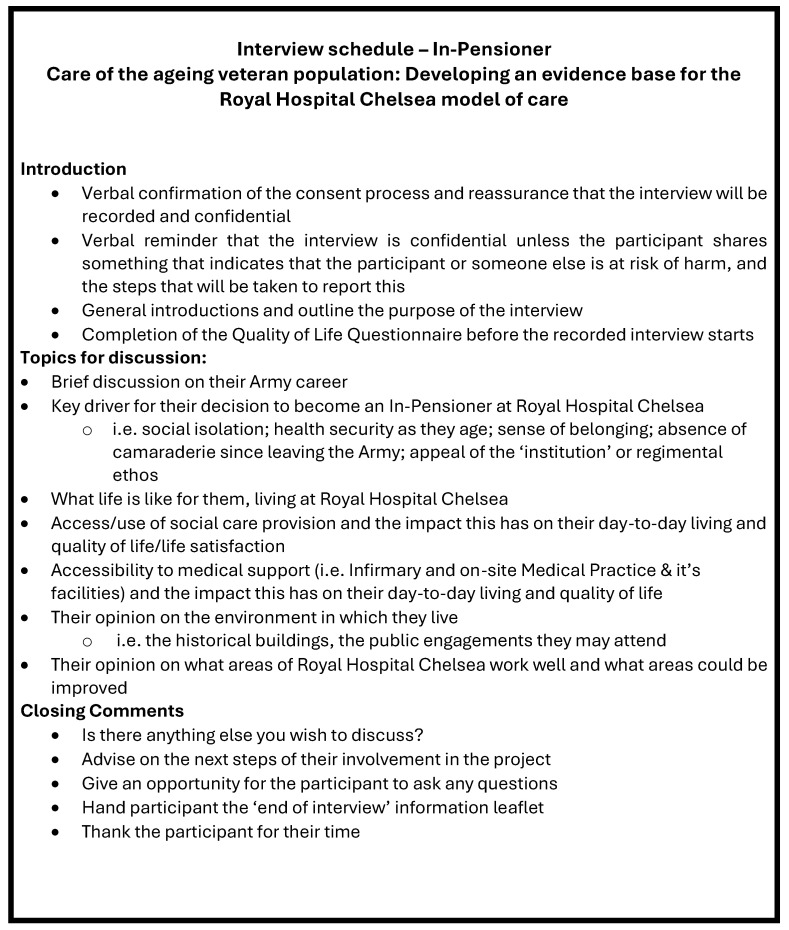
Interview schedule—In-pensioner care of the ageing veteran population: Developing an evidence base for the Royal Hospital Chelsea model of care.

## Data Availability

The datasets presented in this article are not readily available because we have not sought approval from the Royal Hospital Chelsea. Requests to access the datasets should be directed to Dr. Gemma Wilson-Menzfeld, gemma.wilson-menzfeld@northumbria.ac.uk.
